# Supplementary material for: Study of the Experimental and Simulated Vibrational Spectra Together with Conformational Analysis of Thioether Cyanobiphenyl-Based Liquid Crystal Dimers
Source: Int J Mol Sci. 2022 Jul 20;23(14):8005. doi: 10.3390/ijms23148005 (PMC9316788; doi:10.3390/ijms23148005)
Supplement: Supplementary file 1 [file ijms-23-08005-s001.zip › ijms-1820847-supplementary.pdf]

Supplementary Materials

Study of the Experimental and Simulated Vibrational Spectra Together with Conformational Analysis of Thioether Cyanobiphenyl-Based Liquid Crystal Dimers

Antoni Kocot [1], Barbara Loska [1], Yuki Arakawa [2], Georg H. Mehl [3] and Katarzyna Merkel [1,*]

[1] Institute of Materials Engineering, Faculty of Science and Technology, University of Silesia,
ul. 75. Pułku Piechoty, 41-500 Chorzów, Poland; antoni.kocot@us.edu.pl (A.K.); barbara.loska@us.edu.pl (B.L.)

[2] Department of Applied Chemistry and Life Science, Graduate School of Engineering, Toyohashi University of Technology, Toyohashi 441-8580, Japan; arakawa@tut.up

[3] Department of Chemistry, University of Hull, Hull HU6 7RX, UK; g.h.mehl@hull.ac.uk

* Correspondence: katarzyna.merkel@us.edu.pl; Tel.: +48-32-349-7630

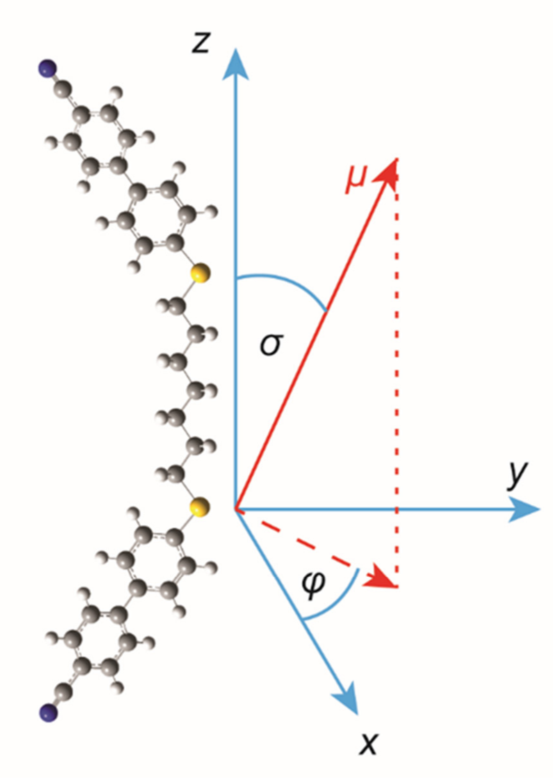

Figure S1. Molecular Frame of Reference: ***z*** – long axis (bowstring), ***x***-axis normal to the bent plane, ***y*** – bow arrow axis, **σ** – polar angle (between transition dipole, **μ**, and the ***z***-axis of the molecule), **φ** is the azimuthal angle that the transition dipole makes with the ***x-z*** plane.

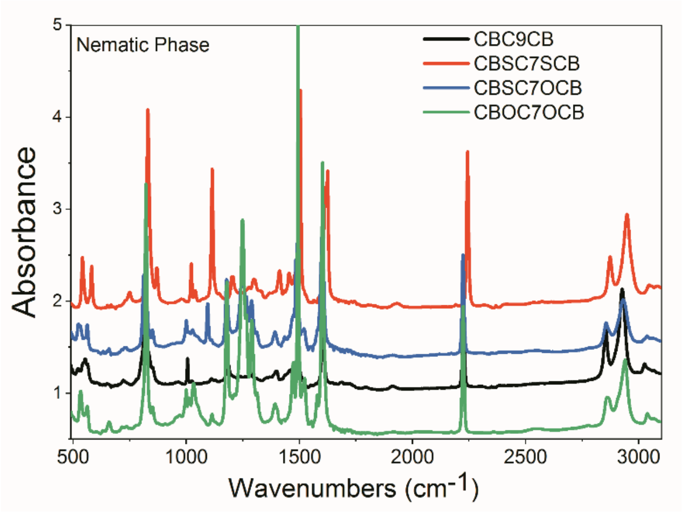

Figure S2. Non-polarized Infrared spectra for all investigated dimers in the nematic phase.

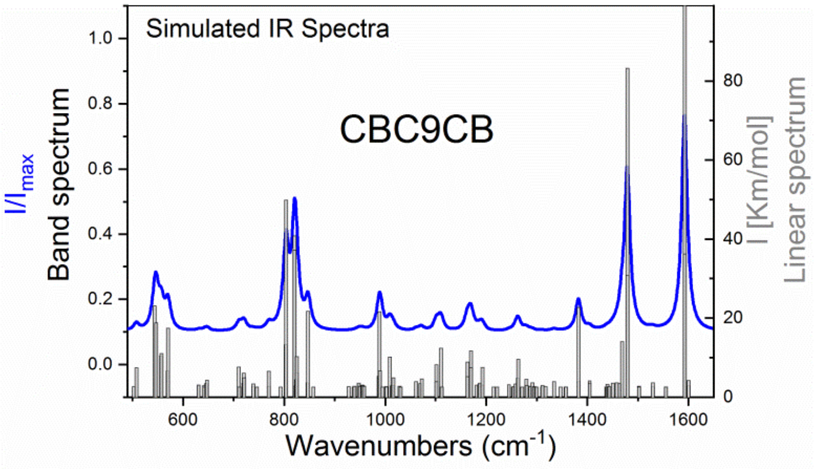

Figure S3. An example comparison of a discrete (linear) spectrum and a band spectrum (extended by the Gaussian function with a half-width of 7 cm^{-1}).

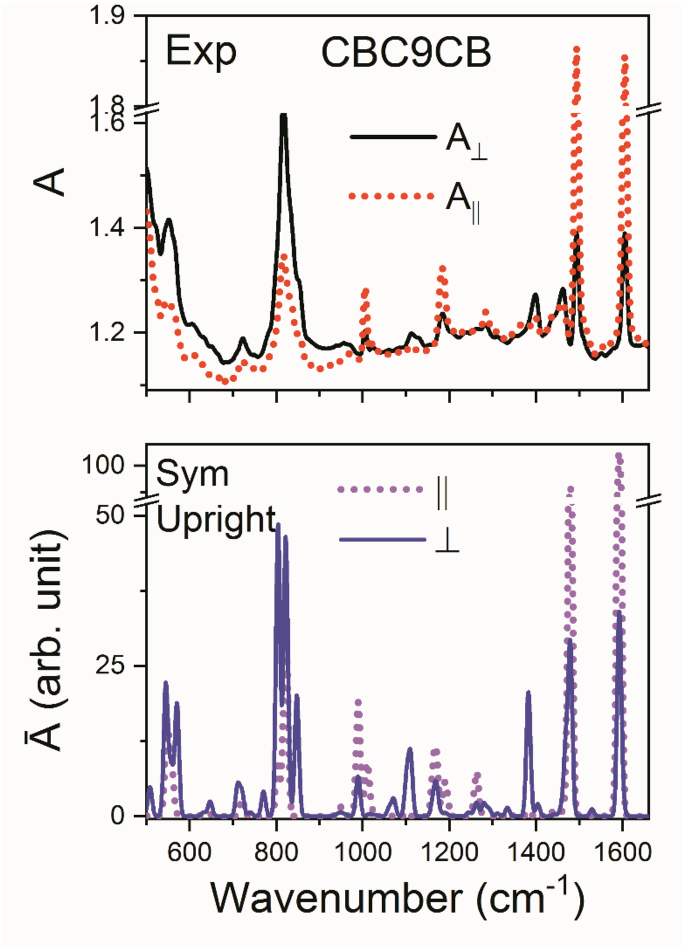

Figure S4. The comparison of the polarized experimental spectrum with the theoretical spectra (B3-LYP/6-311G (d,p)) for the CBC9CB dimer in the region of 500–1650 cm^{-1}. $A_{||}$ – the parallel absorbance component in the z-axis direction of the molecular system, which coincided with the ordering axis of the sample. $A_{\perp}$ – the perpendicular absorbance component was perpendicular to the rubbing direction. Top Figure – experimental spectra of the nematic phase. Bottom Figure – calculated spectra for an upright conformation (dihedral angles C_{Al}-S-C_{Ar}-C_{Ar} = 90°).

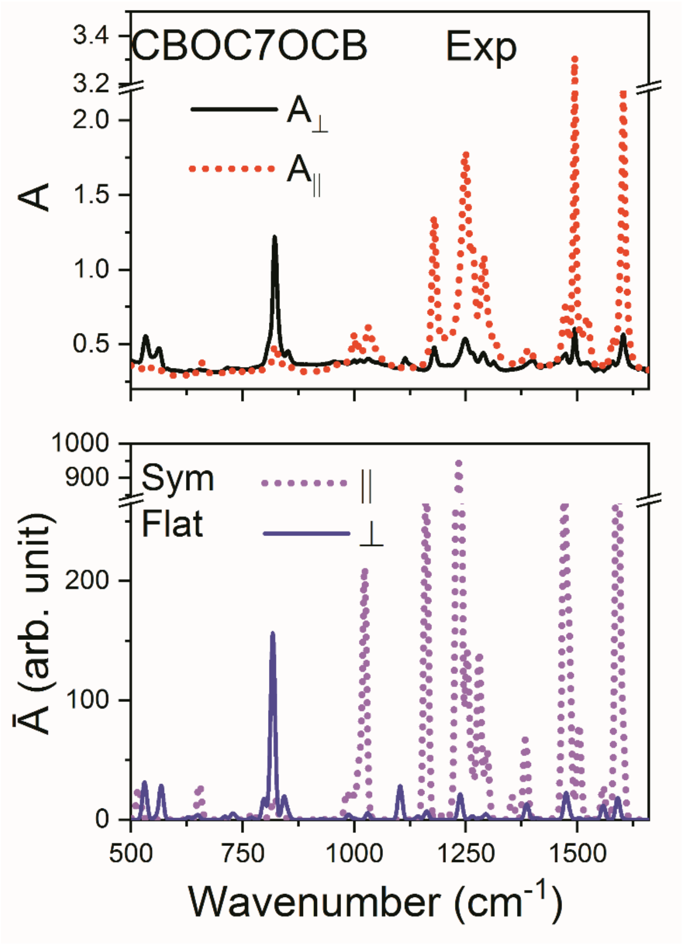

Figure S5. The comparison of the polarized experimental spectrum with the theoretical spectra (B3-LYP/6-311G (d,p)) for the CBOC7OCB dimer in the region of 500–1650 cm^{-1}. $A_{\parallel}$ – the parallel absorbance component in the z-axis direction of the molecular system, which coincided with the ordering axis of the sample. $A_{\perp}$ – the perpendicular absorbance component was perpendicular to the rubbing direction. Top Figure – experimental spectra of the nematic phase. Bottom Figure – calculated spectra for an flat conformation (dihedral angles C_{Al}-O-C_{Ar}-C_{Ar} = 0°).

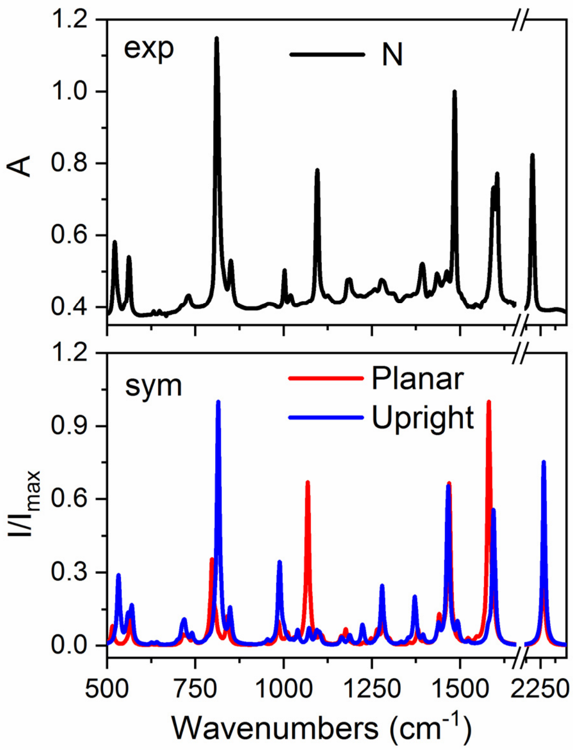

Figure S6. The comparison of the unpolarized experimental spectrum with the theoretical spectra (B3-LYP/6-311G (d,p)) for the CBSC7SCB dimer in the region of 500–2300 cm^{-1}.

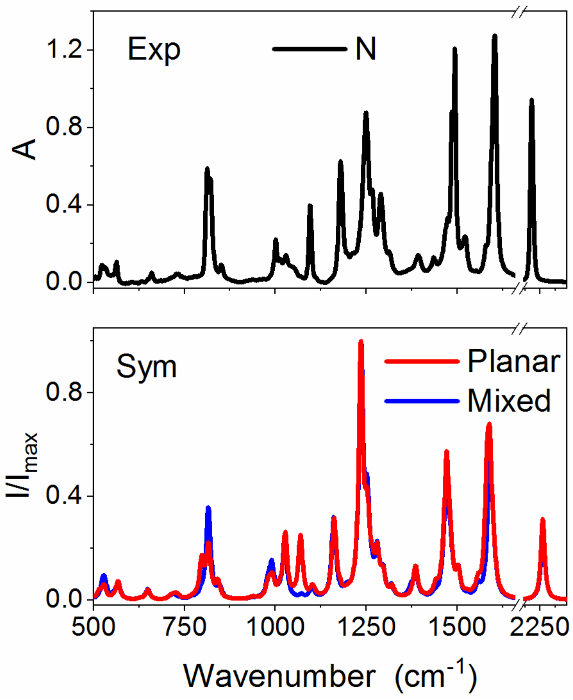

Figure S7. The comparison of the unpolarized experimental spectrum with the theoretical spectra (B3-LYP/6-311G (d,p)) for the CBSC7SCB dimer in the region of 500–2300 cm^{-1}.

Description of abbreviations and symbols contained in tables S1-S4.

Simulated IR Spectra:

Description of conformers: U - upright conformation, F - flat conformation, M - mixed conformation
Linear spectra: ν^* - the frequency of normal vibrations [cm^{-1}], I - intensity [Km / mol]
Band spectrum: ν - vibration frequency expressed in wave numbers [cm^{-1}] scaled with a scaling factor (0.976); I_w - Relative intensity of the bands, ||, ⊥ - parallel and perpendicular component of spectral density, R – dichroism $R = \mu_{\parallel}^2/\mu_{\perp}^2$, μ - direction of the transition dipole moment according to the molecular reference system (Fig. S1), ⊥yz - bent plane of dimer, ⊥xz - perpendicular to the bent plane, || z - long axis of the dimer (bowstring).

Experimental Spectra:

ν - wavenumber [cm-1], I_w - Relative intensity of the bands, || - spectrum with polarizer setting ω = 0 °, ⊥ - spectrum with polarizer setting ω = 90 °, R - dichroism, μ - direction of the transitions dipole moment
Key of vibration assignment: ip – in plane vibration; op – out of plane deformation, br – stretching and deformation vibrations of the ring (benzene ring), s – symmetrical, as – asymmetric, Al. – alkyl chain, Ar – aromatic ring, ν – stretching, γ – deforming out of plane, β – deforming in plane, δ – deforming, vs – very strong, s – strong, m – medium, w – weak, vw – very weak, sh – shoulder.

Table S1. Theoretical and experimental frequencies, dichroism values, relative intensity, direction of the transition dipole moment and approximate band assignments for CBC9CB.

Sym.							Exp					Assignment
Discrete spectrum		Band spectrum										
ν^* [cm^{-1}]	I [$\frac{Km}{mol}$]	ν [cm^{-1}]	I_w ∥	I_w ⊥	R	μ	ν [cm^{-1}]	I_w ∥	I_w ⊥	R	μ	
562,9	20,5	546,4	vw	vw	1,19	∥ z	541,9	vw	w	0,90	⊥	γCC op CB
566,0	16,2											
575,9	7,9	557,0	vw	vw	1,89	∥ z	554,5	vw	w	0,89	⊥	δCN + γCH op CB
576,5	8,5											
589,3	4,1	569,6	vw	vw	0,06	⊥xz	567,0	vw	w	0,91	⊥	γCC op CB + γCH op CB
589,8	14,9											
795,7	0,2	770,7	vw	vw	0,06	⊥yz	781,0	vw	vw	0,96	⊥	βCC ip CB
796,4	4,0											
830,5	10,7	803,6	vw	w	0,33	⊥yz	816,7	w	s	0,91	⊥	γCH op CB
831,1	47,3											
848,5	38,2	821,0	w	w	1,32	∥yz	819,7	s	s	0,82	⊥	
848,7	34,6											
853,3	3,6	-	sh	sh	-	⊥yz	-	sh	sh	-	⊥	γCH op CB(CN) + νCCC skeletal
853,5	7,7											
875,3	1,1	846,1	vw	vw	0,00	⊥yz	851,4	vw	vw	0,92	⊥	
876,0	19,1											
979,6	0,5	950,6	vw	vw	4,67	∥ z	968,1	vw	vw	1,01	∥	νCCC skeletal + γCH op CB
979,6	1,0											
1022,8	2,7	989,2	vw	vw	4,94	∥ z	1005,7	vw	vw	1,07	∥	βCC ip CB, breathing
1022,7	19,0											
1023,0	4,1											
1043,5	7,6	1009,5	vw	vw	78,00	∥ z	1023,1	vw	vw	1,01	∥	νCCC skeletal
1108,6	2,1	1069,5	vw	vw	0,00	⊥yz	1085,7	vw	vw	0,91		νCCC skeletal + βCH ip CB
1139,0	5,7	1102,4	vw	vw	1,13	∥z	1111,8	vw	vw	1,01	∥	βCH ip CB
1139,0	1,4											

Table S1. Continued

Sym.							Exp.					
Discrete spectrum		Band spectrum										Assignment
ν^* [cm^{-1}]	$I \left[\frac{\mathrm{Km}}{\mathrm{mol}}\right]$	ν [cm^{-1}]	I_w ∥	I_w ⊥	R	μ	ν [cm^{-1}]	I_w ∥	I_w ⊥	R	μ	
1148,3	9,8	1109,1	vw	vw	0,03	⊥xz	1127,2	vw	vw	0,98	⊥~~xz~~	βCH ip CB
1149,9	0,0											
1202,1	6,3	1169,1	vw	vw	3,37	∥ z	1184,1	w	vw	1,07	∥ ~~z~~	βCH ip CB
1202,1	2,6											
1209,8	9,1											
1209,8	4,9											
1305,7	2,2	1190,4	vw	vw	17,00	∥ z	1284,4	vw	vw	1,02	∥ ~~z~~	$\gamma_s CH_2$ wagging<br>$\gamma_{as} CH_2$ twisting
1306,0	7,1											
1429,6	1,4	1382,8	vw	vw	0,07	⊥yz	1397,2	vw	vw	0.90	⊥	βCH ip CB
1429,6	20,1											
1514,0	0,9	-	sh	sh	-	⊥yz	-	sh	sh	-	⊥	$\beta_s CH_2$ scissoring
1518,6	11,5											
1529,5	80,5	1478,5	s	vw	4,70	∥ z	1493,6	vs	w	1,34	∥ ~~z~~	νCC br CB
1529,5	28,2											
1646,4	113,4	1591,7	vs	vw	5,75	∥ z	1604,5	vs	w	1,34	∥ ~~z~~	νCC br CB
1646,5	33,7											
2335,4	92,5	2258,9	vs	vw	4,20	∥ z	2224,5	vs	w	1,36	∥ ~~z~~	ν CN
235,4	37,9											
3007,4	31,1	2923,2	vw	vs	0,03	⊥yz	2854,2	m	m	0,93	⊥	$\nu_s CH_2$
3013,3	56,2											
3023,5	185,4											
3054,8	54,3	2965,8	vw	vs	0,00	⊥ xz	2927,4	s	vs	0,88	⊥	$\nu_{as} CH_2$
3067,6	181,4											
3156,3	9,4	3052,8	w	vw	2,18	∥ z	3025,8	w	vw	1,04	∥	νCH CB
3156,3	14,2											
3156,6	32,8											
3156,6	14,3											

Table S2. Theoretical and experimental frequencies, dichroism values, relative intensity, direction of the transition dipole moment and approximate band assignments for CBSC7SCB.

Sym.														Exp.					Assignment
Discrete spectrum				Band spectrum															
Conf. U		Conf. F		Conf. U					Conf. F					ν [cm^{-1}]	I_w		R	μ	
ν [cm^{-1}]	I [Km/mol]	ν [cm^{-1}]	I [Km/mol]	ν [cm^{-1}]	I_w ∥	I_w ⊥	R	μ	ν [cm^{-1}]	I_w ∥	I_w ⊥	R	μ		∥	⊥			
549,9	21,1	533,0	0,0	531,9	w	w	0,96	⊥yz	514,4	vw	w	0,00	⊥xz	521,7	vw	w	0,80	⊥	γCC op CB (S) + δCS
551,6	23,1	533,0	26,1																
579,6	13,7	577,4	2,1	559,9	vw	w	0,13	⊥yz	-	-	-	-	-	551,5	vw	vw	1,02	∥	δCN
589,2	3,9	586,1	24,4	569,6	vw	w	0,20	⊥yz	566,7	vw	w	0,21	⊥xz	562,2	vw	vw	0,85	⊥	γCC op CB + δCN
589,5	18,1	586,2	9,7																
-	-	824,4	112,0	815,2	vs	vs	1,09	∥ z	797,8	vw	vs	0,01	⊥xz	810,9	w	vs	0,55	⊥	γCH op CB
842,7	71,7	-	-						806,5	vw, sh	w, sh	0,01	⊥xz						
842,8	78,7	834,5	24,7																
877,7	20,7	869,8	14,5	849,0	vw	w	0,00	⊥yz	841,3	vw	vw	0,12	⊥yz	851,4	vw	vw	0,89	⊥	γCH op CB + νCCC skel.
877,7	0,2	869,8	24,1																
1022,4	34,9	1018,6	5,6	989,2	m	w	2,15	∥ z	985,4	vw	vw	4,62	∥ z	1002,8	vw	vw	1,35	∥	βCC ip CB, breathing
1022,5	16,1	1019,0	25,9																
-	-	1105,0	173,4	1072,4	vw	vw	6,57	∥ z	1068,5	s	m	3,51	∥ z	1095,4	s	w	1,91	∥	$\nu_{as}C_{Ar}S$ + βCH ip CB
1109,0	8,6	1105,5	49,5																
-	-	1216,4	13,3	-	-	-	-	-	1175,9	vw	vw	1,94	∥ z	1185,1	vw	vw	1,18	∥	βCH ip CB
-	-	1216,8	6,9																
1322,9	36,1	1326,1	32,4	1279,3	m	vw	64,65	∥ z	1282,2	vw	vw	196,24	∥ z	1278,6	vw	vw	1,05	∥	$\gamma_{s}CH_2$ wagging $\gamma_{as}CH_2$ twisting
1418,8	1,8	1427,3	3,9	1372,2	vw	m	0,01	⊥xz	1379,9	vw	vw	0,16	⊥yz	1393,3	vw	vw	0,86	⊥	βCH ip CB
1418,8	28,0	1427,4	17,3																

Table S2. Continued

Sym.															Exp.				
Discrete spectrum				Band spectrum											ν [cm^{-1}]	I_w		R	μ	Assignment
Conf. U		Conf. F		Conf. U					Conf. F										
				ν [cm^{-1}]	I_w		R	μ	ν [cm^{-1}]	I_w		R	μ		∥	⊥			
ν [cm^{-1}]	I $\left[\frac{Km}{mol}\right]$	ν [cm^{-1}]	I $\left[\frac{Km}{mol}\right]$		∥	⊥				∥	⊥								
1497,7	10,2	1490,1	34,8	1438,9	vw	vw	11,10	∥ z	1440,8	vw	vw	29,45	∥ z	1462,8	vw	vw	1,04	∥	$\beta_s CH_2$ scissoring
1516,1	58,1	1517,2	14,8	1466,0	s	m	1,45	∥ z	1468,9	s	m	2,52	∥ z	1484,9	vs	w	2,09	∥	νCC br CB
1516,2	21,5	1519,2	158,1																
1516,5	18,5	1519,6	49,1																
1633,3	6,0	1635,5	265,1	1580,1	vw	vw	14,16	∥ z	1582,0	vs	m	3,86	∥ z	1594,9	m	vw	1,79	∥	νCC br CB
1633,4	0,2	1636,1	66,2																
1649,4	51,3	1650,0	26,6	1594,6	s	m	1,58	∥ z	1595,6	vw	vw	1,88	∥ z	1605,5	s	w	1,83	∥	
1649,4	32,6	1650,0	14,7																
2336,9	65,8	2335,2	103,1	2259,9	vs	s	1,39	∥ z	2257,9	m	w	2,35	∥ z	2224,5	s	w	1,97	∥	νCN
2336,9	48,0	2335,3	53,9																
3025,2	32,9	3027,9	13,7	2925,2	vw	m	0,04	⊥yz	2926,1	vw	vw	0,13	⊥yz	2854,2	vw	vw	0,89	⊥	$\nu_s CH_2$
3044,9	55,3	3040,7	60,2	2944,5	vw	s	0,11	⊥yz	2940,6	vw	m	0,07	⊥yz						
3065,0	29,0	30,7	21,7	2963,9	vw	w	0,00	⊥xz	2966,8	vw	vw	0,00	∥ z						
3097,8	52,3	3095,7	59,3	2995,8	vw	s	0,00	⊥xz	2993,8	vw	m	0,00	∥ z	2928,4	vw	w	0,84	⊥	$\nu_{as} CH_2$

Table S3. Theoretical and experimental frequencies, dichroism values, relative intensity, direction of the transition dipole moment and approximate band assignments for CBSC7OCB.

Sym.														Exp.					Assignment
Discrete spectrum				Band spectrum															
Conf. M		Conf. F		Conf. M					Conf. F					ν [cm^{-1}]	I_w		R	μ	
				ν [cm^{-1}]	I_w		R	μ	ν [cm^{-1}]	I_w		R	μ		‖	⊥			
ν [cm^{-1}]	I [Km/mol]	ν [cm^{-1}]	I [Km/mol]		‖	⊥				‖	⊥								
542,8	23,9	532,6	12,9	-	-	-	-	-	515,4	vw	vw	1,73	⊥xz	522,6	vw	w	0,31	⊥	γCC op CB(S) + δCS
547,5	14,9	544,6	10,6	528,0	vw	vw	0,49	⊥xz	528,9	vw	w	0,50	⊥xz	532,3	vw	w	0,29	⊥	γCC op CB(O) + δCO
551,1	11,7	547,8	15,3																
586,3	15,9	586,4	17,1	567,6	vw	w	0,12	⊥xz	567,6	vw	vw	0,08	⊥xz	563,1	vw	w	0,31	⊥	γCC op CB +δCN
589,6	11,2	586,8	15,1																
671,6	17,5	672,2	17,2	649,8	vw	vw	5,73	‖ z	649,8	vw	vw	5,97	‖ z	659,5	vw	vw	3,98	‖	ν_sCOC + βCC ip CB
823,4	10,0	823,6	36,2	797,8	vw	s	0,03	⊥xz	797,8	vw	vw	0,08	⊥xz	812,9	vw	vs	0,29	⊥	γCH op CB
-	-	825,7	32,6																
843,0	41,2	-	-	816,1	vw	vs	0,08	-	816,1	vw	vs	0,24	⊥xz	821,5	vw	vs	0,31	⊥	ν_sCOC + γCH op CB
843,4	110,5	-	-																
-	-	843,9	73,6																
-	-	845,2	15,3																
871,8	10,1	870,2	20,0	843,2	vw	w	0,17	⊥yz	841,3	vw	vw	0,13	-	850,5	vw	vw	0,56	⊥	γCH op CB + νCCC skeletal
878,3	10,8	872,7	10,0																
1022,4	28,2	-	-	989,2	vw	vw	30,18	‖ z	989,2	vw	vw	9,35	‖ z	1000,9	vw	vw	4,07	‖	βCC ip CB, breathing
1025,3	28,4	1025,9	30,6																
1061,0	103,5	1062,2	110,1	1026,0	w	vw	69,41	‖ z	1027,0	w	vw	133,42	‖ z	1028,8	vw	vw	3,47	‖	$\nu_{as}C_{Al}O$ +βCH ip CB
1062,3	1,4	1062,8	4,7																
1081,2	0,4	1084,8	1,2	1045,3	vw	vw			1045,3	vw	vw			1051	sh	sh			
-	-	1105,7	111,3	-	-	-	-	-	1069,5	vw	vw	5,70	‖ z	1095,4	w	vw	4,41	‖	$\nu_{as}C_{Ar}S$ + βCH ip CB
-	-	1106,6	0,6																
1199,7	108,4	1200,8	104,1	1160,4	w	vw	12,29	‖ z	1161,4	w	vw	11,07	‖ z	1179,3	m	vw	4,69	‖	βCH ip CB
1202,5	31,7	1202,8	31,9																
1277,8	424,4	1277,5	44,2	1235,8	vs	w	19,65	‖ z	1235,8	vs	vw	15,74	‖ z	1249,7	s	w	5,06	‖	$\nu_{as}C_{Ar}O$ + βCC ip CB
1295,0	116,8	1295,5	118,1	1251,3	w	vw	34,43	‖ z	1251,3	w	vw	23,75	‖ z	1266,0	w,sh	vw,sh	4,36	‖	
1296,3	29,5	1297,0	7,7																

Sym.														Exp.					Assignment
Discrete spectrum				Band spectrum															
Conf. M		Conf. F		Conf. M					Conf. F						I_w				
ν [cm^{-1}]	I [Km/mol]	ν [cm^{-1}]	I [Km/mol]	ν [cm^{-1}]	I_w ‖	I_w ⊥	R	μ	ν [cm^{-1}]	I_w ‖	I_w ⊥	R	μ	ν [cm^{-1}]	‖	⊥	R	μ	
1323,6	73,0	1323,7	71,3	1279,3	vw	vw	89,31	‖ z	1279,3	vw	vw	102,56	‖ z	1290,2	w	vw	4,90	‖	$\gamma_s CH_2$ wagging $\gamma_{as} CH_2$ twisting
1328,5	0,4	1330,2	0,2																
-	-	1341,8	23,8	1294,8	vw	vw	11,59	‖ z	1294,8	vw	vw	9,88	‖ z	1313,3	vw	vw	3,52	‖	
1341,3	28,9	-	-																
1419,1	15,1	1427,6	10,4	1385,7	vw	vw	3,21	‖ z	1385,7	vw	vw	4,50	⊥yz	1393,3	vw	vw	1,33	⊥	βCH ip CB
1431,3	21,6	1431,8	12,5																
1433,6	26,3	1433,9	38,2																
1486,7	5,0	1489,1	15,4	1439,9	vw	vw	99,80	‖ z	1439,9	vw	vw	30,21	‖ z	1473,4	w	vw	3,52	‖	
1516,5	48,9	1519,9	110,0	1471,8	m	m	5,31	‖ z	1471,8	w	w	4,87	‖ z	1485,9	s	w	5,13	‖	νCC br CB
1522,2	131,6	1522,4	129,8																
1531,8	70,6	1531,9	67,5	1481,4	vw	vw	9,28	‖ z	1481,4	w	vw	8,47	‖ z	1493,6	vs	w	5,37	‖	
1554,4	42,0	1554,1	41,9	1502,7	vw	vw	23,71	‖ z	1502,7	vw	vw	16,51	‖ z	1521,6	vw	vw	5,91	‖	
1610,2	20,7	1610,3	21,9	1557,8	vw	vw	1,99	-	1557,8	vw	vw	1,65	-	1579,4	vw	vw	3,04	‖	
-	-	1636,0	178,2	1589,7	m	w	9,02	‖ z	1589,7	m	w	8,18	‖ z	1602,6	vs	w	4,98	‖	νCC br
1643,8	232,6	1643,9	212,5																
1649,5	39,1	1650,0	19,6																
1655,9	37,6	1655,8	37,3																
2334,6	74,4	2334,8	75,9	2258,9	w	w	4,65	‖ z	2257,9	w	w	3,69	‖ z	2224,5	s	w	5,52	‖	νCN
2337,0	75,4	2335,3	70,5																
2989,3	22,8	2988,7	23,2	2891,3	vw	vw	1,40	⊥yz	2890,4	vw	vw	1,60	-	2856,1	vw	w	0,55	⊥	$\nu_s CH_2$
3003,4	14,3	3003,4	15,4	2903,9	vw	vw	0,05	⊥yz	2903,9	vw	vw	0,04	⊥yz						
3022,0	10,0	3024,6	3,4	2932,9	vw	s	0,06	⊥xz	2926,1	vw	w	0,06	⊥xz	2931,3	vw	m	0,57	⊥	$\nu_{as} CH_2$
3026,0	20,8	3025,3	21,1																
3033,7	33,8	3033,5	19,4	2942,6	vw	w	0,01	⊥xz	2936,8	vw	w	0,06	⊥xz	3040,3	vw	vw	1,54	‖	νCH CB
3044,7	34,0	3039,2	46,1																
3077,7	52,4	3077,7	47,5	2976,4	vw	m	0,00	⊥xz	2976,4	vw	m	0,00	⊥xz	-	-	-	-	⊥	
3097,5	31,1	3094,0	37,6	2994,8	#N/D	w	0,00	⊥xz	2990,9	vw	vw	0,00	⊥xz	-	-	-	-	⊥	

Table S4. Theoretical and experimental frequencies, dichroism values, relative intensity, direction of the transition dipole moment and approximate band assignments for CBOC7OCB.

Sym.							Exp.					Assignment
Discrete spectrum		Band spectrum										
ν $[cm^{-1}]$	I $\left[\frac{Km}{mol}\right]$	ν $[cm^{-1}]$	I_w ∥	I_w ⊥	R	μ	ν $[cm^{-1}]$	I_w ∥	I_w ⊥	R	μ	
547,9	30,3	529,9	vw	w	0,05	⊥xz	532,3	vw	w	0,65	⊥	γCC op CB (O) + δCO
548,6	1,0											
586,7	1,9	567,6	vw	vw	0,06	⊥xz	562,2	vw	vw	0,73	⊥	γCC op CB + δCN
586,7	28,3											
675,0	30,0	652,7	vw	vw	9,89	∥ z	658,6	vw	vw	1,15	∥	βCC ip CB
824,6	17,7	797,8	vw	vw	0,10	⊥xz	806,1	vw	vw	0,72	⊥	γCC op CB
827,5	2,1											
845,3	143,5	817,1	vw	vs	0,10	⊥xz	821,5	vw	vs	0,40	⊥	ν_sCOC + γCH op CB
547,7	10,6											
870,9	18,8	841,3	vw	vw	0,11	⊥yz	851,4	vw	vw	0,81	⊥	γCH op CB + νCCC skeletal
872,3	2,3											
1016,3	0,5	983,4	vw	vw	6,92	∥ z	999,9	vw	vw	1,45	∥	βCC ip CB, breathing
1016,5	23,3											
1029,6	18,9	996,0	vw	vw	13,85	∥ z	1012,5	vw	vw	1,29	∥	νCCC skeletal + βCH ip CB
1044,7	46,3	1011,5	vw	vw	412,14	∥ z	-	sh	sh	-	-	$\nu_{as}C_{Al}O$ +βCH ip CB
1058,3	210,3	1023,1	w	vw	123,42	∥ z	1032,7	vw	vw	1,53	∥	
1139,8	0,1	1103,3	vw	vw	0,04	⊥yz	1113,7	vw	vw	0,89	⊥	νCCC skeletal
1139,9	13,9											
1140,7	1,2											
1141,2	14,5											
1200,2	235,6	1161,4	w	vw	39,96	∥ z	1179,3	m	vw	2,82	∥	βCH ip CB
1200,7	4,4											
1203,3	54,1											
1203,3	3,3											
1276,3	945,5	1233,9	vs	vw	58,23	∥ z	1248,7	s	w	3,33	∥	$\nu_{as}C_{Ar}O$ +βCH ip CB
1279,9	20,8											

Table S4. Continued

Sym.							Exp.					Assignment
Discrete spectrum		Band spectrum										
ν [cm^{-1}]	I $\left[\frac{\text{Km}}{\text{mol}}\right]$	ν [cm^{-1}]	I_w		R	μ	ν [cm^{-1}]	I_w		R	μ	
			‖	⊥				‖	⊥			
1295,5	137,7	1252,3	vw	vw	406,28	‖ z	1266,0	w	vw	2,55	‖	
1296,4	0,1											
1323,3	238,5	1279,3	vw	vw	92,85	‖ z	1290,2	w	vw	2,47	‖	$\gamma_s CH_2$ wagging<br>$\gamma_{as} CH_2$ twisting
1323,3	3,9											
1337,3	15,6	1295,8	vw	vw	12,17	‖ z	1313,3	vw	vw	1,46	‖	
1339,9	3,3											
1342,1	47,3											
1343,7	0,6											
1430,2	50,4	1383,8	vw	vw	5,88	‖ z	1392,4	vw	vw	1,23	‖	βCH ip CB
1432,5	8,4											
1433,4	20,3											
1435,5	5,2											
1520,5	268,1	1470,8	w	vw	16,24	‖ z	1492,6	vs	w	4,02	‖	νCC br
1524,4	21,5											
1531,8	145,4											
1532,8	4,1											
1554,6	80,1	1502,7	vw	vw	52,00	‖ z	1523,5	vw	vw	1,81	‖	νCC br + $\beta_s CH_2$ + $\nu_{as} C_{Ar}O$
1555,1	1,3											
1610,4	28,6	1556,9	vw	vw	2,43	‖ z	1578,5	vw	vw	1,40	‖	
1610,7	11,9											
1643,7	422,3	1589,7	m	vw	23,25	‖ z	1602,6	vs	w	3,92	‖	νCC br
164,5	18,4											
1656,0	72,4											
1656,4	0,6											
2334,7	134,1	2257,9	vw	vw	11,54	‖ z	2223,5	m	vw	2,79	‖	νCN
2334,7	11,7											

Table S4. Continued

Sym.								Exp.					Assignment
Discrete spectrum		Band spectrum											
ν [cm^{-1}]	I $\left[\frac{\text{Km}}{\text{mol}}\right]$	ν [cm^{-1}]	I_w		R	μ		ν [cm^{-1}]	I_w		R	μ	
			‖	⊥					‖	⊥			
2988,7	20,0	2891,3	vw	vw	1,21	‖ z		2863,8	vw	vw	2,84	‖	ν_sCH_2
2989,4	25,1												
3002,4	28,0	2902,9	vw	vw	0,03	⊥yz							
3026,2	46,4	2927,1	vw	w	0,00	⊥xz		-	sh	sh	-	⊥	$\nu_{as}CH_2$<br>ν_sCH_2
3026,8	2,7												
3037,3	55,8	2935,8	vw	w	0,00	⊥yz							
3082,5	92,6	2980,3	vw	m	0,00	⊥xz		2939,0	vw	w	0,73	⊥	$\nu_{as}CH_2$
